# Supplementary material for: Comparative Profiling of Volatile Compounds and Fatty Acids in Pomegranate Seed Oil: Soxhlet vs. CO2/IPA Extraction for Quality and Circular Bioeconomy Goals
Source: Foods. 2025 Aug 25;14(17):2951. doi: 10.3390/foods14172951 (PMC12428489; doi:10.3390/foods14172951)
Supplement: Supplementary file 1 [file foods-14-02951-s001.zip › foods-3819452-supplementary.pdf]

Comparative Profiling of Volatile Compounds and Fatty Acids in Pomegranate Seed Oil: Soxhlet *vs* CO_2/IPA Extraction for Quality and Circular Bioeconomy Goals

Caterina Fraschetti[1], Antonello Filippi[1], Antonia Iazzetti[2], Giancarlo Fabrizi[1], Francesco Cairone[1]*, Stefania Cesa[1]

[1]Department Chemistry and Technologies of Drug, University "La Sapienza" of Rome, P.le Aldo Moro 5, 00185 Rome, Italy

[2]Department of Basic Biotechnological Sciences, Intensivological and Perioperative Clinics, Catholic University of the Sacred Heart, L.go F. Vito 1, 00168 Rome, Italy

*francesco.cairone@uniroma1.it

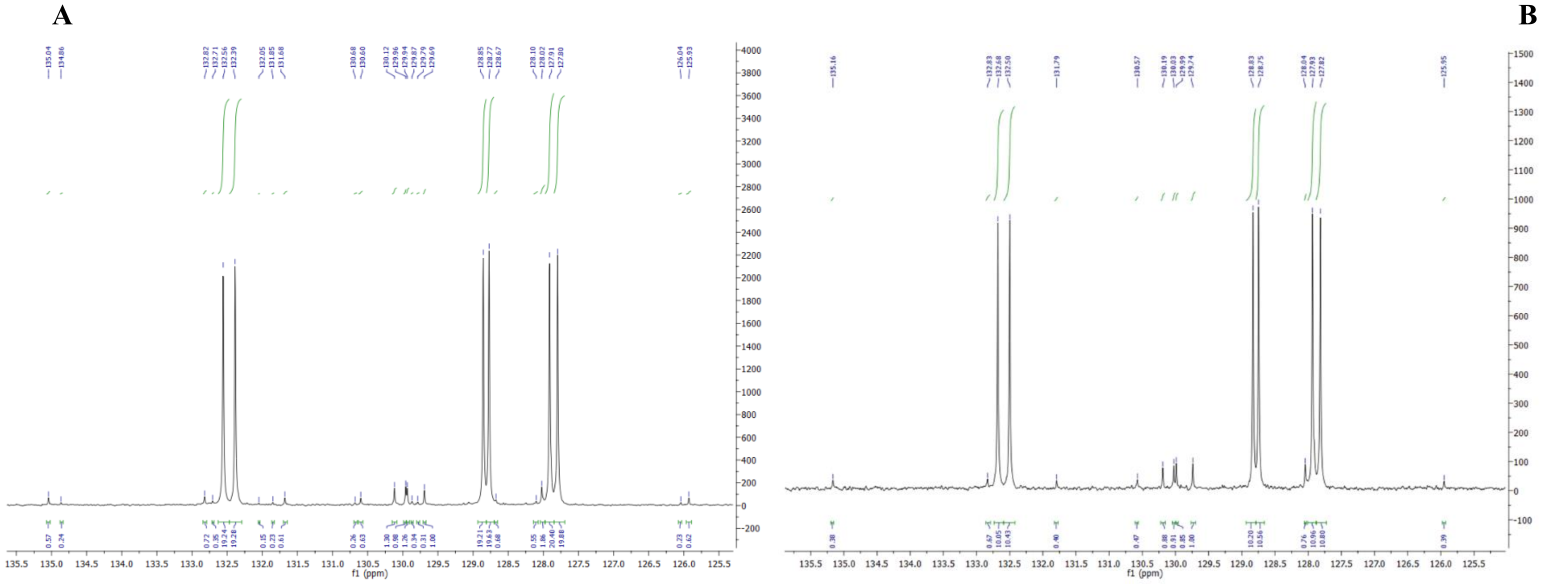

Figure S1. Panel A: ^{13}C-NMR spectrum of $G1_{FAME}$ samples; Panel B: ^{13}C-NMR spectrum of $R1_{FAME}$ samples.

A B

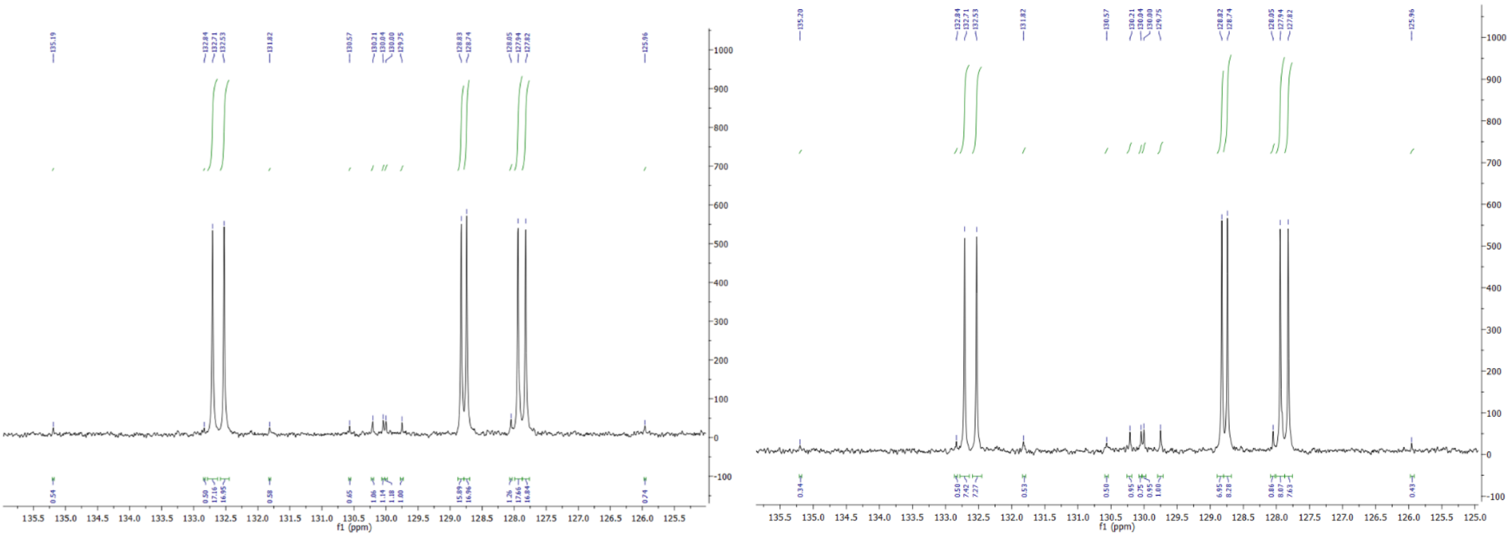

Figure S2. Panel A: ^{13}C-NMR spectrum of G2$_{FAME}$ samples; Panel B: ^{13}C-NMR spectrum of R2$_{FAME}$ samples.

Table S1. GC-MS analysis of FAMEs fraction in pomegranate Soxhlet extracts (**G1$_{FAME}$** and **R1$_{FAME}$**)

FA precursor			Area %	RI[a]	RIL[b]
		**R1$_{FAME}$**			
**IUPAC**	**Common**	**Composition**			
hexadecanoic	palmitic	C16:0	1.5	1938	1925
9-cis,12-cis-octadecadienoic	linoleic	C18:2	2.6	2108	2093
cis-9-octadecenoic	oleic	C18:1	7.2	2112	2104
octadecanoic	stearic	C18:0	1.4	2136	2127
(9Z,11E,13Z)-octadeca-9,11,13-trienoic	punicic	C18:3	81.1	2248	2237
(9Z,11E,13E)-octadeca-9,11,13-trienoic	α-eleostearic	C18:3	6.2	2265	2257
		**G1$_{FAME}$**			
hexadecanoic	palmitic	C16:0	1.8	1938	1925
9-cis,12-cis-octadecadienoic	linoleic	C18:2	2.5	2108	2093
cis-9-octadecenoic	oleic	C18:1	5.0	2112	2104
trans-9-octadecenoic	elaidic	C19:1	0.3	2117	
octadecanoic	stearic	C18:0	1.3	2136	2127
(9Z,11E,13Z)-octadeca-9,11,13-trienoic	punicic	C18:3	84.8	2249	2237
(9Z,11E,13E)-octadeca-9,11,13-trienoic	α-eleostearic	C18:3	4.3	2265	2257

[a.] Experimental retention index; [b] Literature Retention index

Table S2. GC-MS analysis of FAMEs fraction in pomegranate supercritical CO_2 extracts (**G2**$_{FAME}$ and **R2**$_{FAME}$)

**FA precursor**			**Area %**	**RI**[a]	**RIL**[b]
		**R2**$_{FAME}$			
**IUPAC**	**Common**	**Composition**			
hexadecanoic	palmitic	C16:0	1.6	1938	1925
9-cis,12-cis-octadecadienoic	linoleic	C18:2	3.2	2108	2093
cis-9-octadecenoic	oleic	C18:1	8.3	2113	2104
octadecanoic	stearic	C18:0	1.7	2136	2127
(9Z,11E,13Z)-octadeca-9,11,13-trienoic	punicic	C18:3	80.3	2248	2237
(9Z,11E,13E)-octadeca-9,11,13-trienoic	α-eleostearic	C18:3	4.9	2265	2257
		**G2**$_{FAME}$			
hexadecanoic	palmitic	C16:0	2.0	1938	1925
9-cis,12-cis-octadecadienoic	linoleic	C18:2	2.6	2108	2093
cis-9-octadecenoic	oleic	C18:1	4.7	2112	2104
octadecanoic	stearic	C18:0	1.3	2136	2127
(9Z,11E,13Z)-octadeca-9,11,13-trienoic	punicic	C18:3	84.7	2249	2237
(9Z,11E,13E)-octadeca-9,11,13-trienoic	α-eleostearic	C18:3	4.7	2265	2257

[a.] Experimental retention index; [b] Literature Retention index
